# QTL analyses of temporal and intensity components of home-cage activity in KJR and C57BL/6J strains

**DOI:** 10.1186/1471-2156-10-40

**Published:** 2009-07-29

**Authors:** Juzoh Umemori, Akinori Nishi, Arimantas Lionikas, Takayuki Sakaguchi, Satoshi Kuriki, David A Blizard, Tsuyoshi Koide

**Affiliations:** 1 Mouse Genomics Resource Laboratory, National Institute of Genetics, Mishima, Shizuoka 411-8540, Japan; 2 Department of Genetics, The Graduate University for Advanced Studies (SOKENDAI), Hayama, Kanagawa, Japan; 3 School of Medical Sciences, College of Life Sciences and Medicine, University of Aberdeen, Aberdeen, UK; 4 Center for Developmental and Health Genetics, Pennsylvania State University, PA, USA; 5 Department of Mathematical Analysis and Statistical Inference; Statistical Genome Diversity Research Group, Prediction and Knowledge Discovery Research Center, The Institute of Statistical Mathematics, Tokyo, Japan

## Abstract

**Background:**

A variety of mouse strains exhibit diversity in spontaneous activity consistent with an important genetic contribution. To date, many studies have defined spontaneous home-cage activity as total distance or total counts of activity within a test period. However, spontaneous activity is, in fact, a composite of elements of 'temporal' and 'intensity' that is similar to 'velocity'. Here, we report on quantitative trait loci for different components of spontaneous activity, an important step towards dissection of the underlying genetic mechanisms.

**Results:**

In the analysis of total home-cage activity (THA) after habituation in female mice, KJR strain exhibit higher activity than C57BL/6J (B6). In this study, THA was partitioned into two components: active time (AT) was an index of the 'temporal element' of THA, average activity during active time (AA) was an index of 'intensity'. Correlation analysis using B6xKJR F_2 _female mice indicated that AA is a major component of THA, whereas AA and AT were associated to a lesser degree. To explore the genetic basis of the activity differences, we conducted quantitative trait loci (QTL) analysis on data of THA and its components, AT and AA. Three significant QTL affecting variation of different components of home cage activity were identified, two linked QTL *Hylaq1 *and *Hylaq2 *on Chr 2, and *Hylaq3 *on Chr 10. Chromosomal positions of these QTL were previously implicated in locomotor activity (Chr 2) or open-field ambulation (Chr 10). The results indicated that *Hylaq1 *influences AT, *Hylaq2*, AA, while *Hylaq3 *is associated with both AA and AT.

**Conclusion:**

Through this study, we found that variation in total home cage activity over a 3 day period is affected by variation in active time and intensity of activity. The latter two variables are distinct components of home cage activity with only partially overlapping genetic architecture.

## Background

Spontaneous activity is a key feature of an animal's interaction with its environment reflecting a variety of metabolic, physiological and neurological processes. It has been reported that many laboratory strains of rodents exhibit variation in locomotor activity that are influenced by genetic factors. For example, Plomin et al. identified quantitative trait loci (QTLs) related to locomotor activity in the open field using Recombinant inbred mouse strains (BXD) [1]. Several other genetic studies focused on differences of the open-field activity between various kind of mouse strains and found many QTLs [2-7]. In rats, Hendley et al. established a Wistar-Kyoto hyperactive (WKHA) strain that exhibits increased spontaneous activity in a novel environment [8]. A subsequent genetic study clarified QTL related to open-field activity in WKHA [9].

However, ambulation or locomotion in open-field often reflects motivational and situational influences [10-13]. From this point of view, measuring home-cage activity is more suitable for estimating spontaneous or general activity, but there are not many reports on QTL related to spontaneous activity in a home cage [14]. Koyner et al. found QTL related to basal activity on Chr 1, 5 and 9 using an arena apparatus covered with standard laboratory bedding that mimicked the home-cage environment [15]. By conducting genetic analysis using similar apparatus, QTLs related to basal activity were identified on Chr 1, 9 and 19 [16]; Chr 1, 2 and 6 [17]; Chr 4, 8, 11, 14, 18 and X [5]; and Chr 1 [18]. Recently, QTL mapping was conducted using automated home-cage measuring apparatus and mapped a locus related to spontaneous activity on Chr 1 [19,20].

Previously, our laboratory conducted multi-phenotype behavioral characterization using a series of inbred strains derived from wild mice in addition to commonly used laboratory strains [21]. The results showed great diversity among strains in terms of the behavioral patterns, including spontaneous locomotor activity in the home cage. In particular, the activity of the KJR/Ms (KJR) strain was significantly higher than that of BLG2 and C57BL/6J (B6). Linkage analysis in the KJR and BLG2 backcross showed that two loci, *Loco1 *on Chr 3 and *Loco2 *on Chr17 influenced spontaneous locomotor activity in the home-cage [22]. However, the actual level of spontaneous locomotor activity during a defined period of time can be achieved by either short bursts of rapid movement or by steady, slow movement throughout the period, furthermore, the frequency of the bursts of activity may also vary. In fact, in most instances both 'temporal' and 'intensity' elements contribute to total activity to varying degrees. Importantly, each of those elements is likely to reflect the function distinct biological mechanisms and recent reports suggested that the genetic variation can differentially influence temporal and velocity (intensity) phenotypes [19]. For instance, the pattern of the bursts of activity is determined by CNS mechanisms influencing sleep and activity, whereas the intensity of activity might be an outcome of neuromuscular function. Therefore, in the present paper we attempted extract additional information about the mechanism contributing to spontaneous home cage activity by partitioning it into two components.

The KJR strain is derived from wild mice captured in Kojuri, Korea [21], and belongs to the musculus subspecies group that is widely distributed from east Europe to Asia. KJR mice have retained some aspects of the wild phenotype, e.g. quick movement, pronounced inter-male aggressive behavior and high emotionality. In particular, KJR mice move quickly for long periods, resulting in high counts in the three-day measurement of spontaneous locomotor activity [22].

B6 is derived from European fancy mice and belongs to the domesticus subspecies group that is distributed in West Europe [23,24]. Compared to KJR, B6 shows typical features of laboratory mice, e.g. slow movement and low-impulsive behavior. In addition, B6 showed intermediate level total home-cage activity among the tested strains, but this was significantly lower than that of KJR [21]. Thus, KJR and B6 are hypothesized to have different genetic mechanisms involved in spontaneous activity in the home cage.

In the present study we aimed to identify the genetic architecture underlying temporal and intensity components of home cage activity using phenotypically divergent inbred strain.

## Methods

### Mouse strains and maintenance

C57BL/6J (B6) and KJR/Ms (KJR) inbred strains were maintained in the animal facility at the National Institute of Genetics (NIG), Mishima, Japan. KJR was established as an inbred strain after 20 generations of brother-sister mating [21]. For genetic mapping, F_2 _populations (B6xKJR F_2_) were generated by an intercross of the F1 mice (B6xKJR F_1_). Based on the previous report in which a significant difference in home-cage activity was observed between B6 and KJR in females [21], 274 female B6xKJR F_2 _mice aged between 8 and 12 weeks were generated and used to measure activities in the home-cage. All mice were maintained at NIG on a 12 h/12 h light/dark cycle with lights coming on at 8:00 a.m. Temperature was maintained at 23 ± 2°C with humidity at 50 ± 10%. Mice were fed a pelleted diet (CE-2; CLEA Japan Inc, Tokyo, Japan) ad libitum, and autoclaved tap water. Mice were maintained according to NIG guidelines, and all procedures were carried out with approval (No. 18-18 and 19-6) from our institutional animal care and use committee.

### Measurement and analysis of spontaneous activity in home cage

The spontaneous activity in the habituated home cage was measured according to the method described previously [21]. The spontaneous home-cage activity was assessed for individual mice with an infrared sensor, AB-system 24 (Neuroscience Co. Ltd., Tokyo, Japan) for a 4-day period, with the first day used for habituation. The photo beam counts were collected for each minute from 08:00 a.m. of the second day to 08:00 a.m. of the fifth day for each mouse. Total spontaneous home-cage activity (THA) was the index of total activity in the home cage and calculated as the sum of total beam counts across the three days.

### Calculation of heritability

Broad-sense heritability (H^2^) is the fraction of the total phenotypic variance (V_T_) that is due to genetic differences among individuals in a population (V_G_) and calculated as follows;



In this study, V_T _was calculated as a variance of B6xKJR F_2 _population, and environmental variance (V_E_) was the variance among the B6xKJR F_1 _population (N = 11). Thus, V_G _was the difference between V_T _and V_E_.

### Genotyping using microsatellite polymorphisms

Genomic DNA of each mouse was isolated from the tail by using an automatic nucleic acid isolation system, NA-2000 (KURABO, Osaka, Japan). A total 135 polymorphic microsatellite markers were chosen for genome-wide scanning of 274 B6xKJR F_2 _progeny. Microsatellite markers were spaced between 5 to 20 cM, and determined by the method as described previously [25]. Microsatellite markers used for B6xKJR F_2 _progeny: *D1Mit296, D1Mit318, D1Mit414, D1Mit132, D1Mit365, D1Mit309, D1Mit16, D2Mit2, D2Mit120, D2Mit203, D2Mit8, D2Mit9, D2Mit126, D2Mit30, D2Mit208, D2Mit423, D2Mit22, D2Mit55, D2Mit29, D2Mit265, D2Mit200, D3Mit149, D3Mit4, D3Mit51, D3Mit216, D3Mit147, D3Mit163, D4Mit235, D4Mit236, D4Mit288, D4Mit153, D4Mit12, D4Mit127, D4Mit254, D5Mit48, D5Mit4, D5Mit6, D5Mit403, D5Mit242, D5Mit51, D6Mit83, D6Mit316, D6Mit102, D6Mit287, D6Mit25, D7Mit191, D7Mit155, D7Mit69, D7Mit194, D7Mit31, D7Mit37, D7Mit66, D7Mit9, D7Mit108, D7Mit12, D8Mit1, D8Mit293, D8Mit69, D8Mit240, D8Mit242, D8Mit200, D8Mit13, D9Mit251, D9Mit23, D9Mit260, D9Mit10, D9Mit182, D9Mit24, D9Mit279, D9Mit121, D10Mit106, D10Mit5, D10Mit15, D10Mit186, D10Mit117, D10Mit70, D10Mit150, D10Mit73, D10Mit295, D10Mit180, D10Mit237, D10Mit145, D10Mit103, D11Mit23, D11Mit28, D11Mit327, D11Mit145, D11Mit214, D12Mit37, D12Mit147, D12Mit5, D12Mit118, D13Mit14, D13Mit88, D13Mit126, D13Mit262, D13Mit35, D14Mit2, D14Mit233, D14Mit102, D14Mit160, D14Mit265, D14Mit107, D15Mit226, D15Mit267, D15Mit111, D15Mit5, D15Mit121, D15Mit156, D16Mit34, D16Mit3, D16Mit65, D16Mit203, D17Mit164, D17Mit11, D17Mit36, D17Mit139, D17Mit129, D18Mit19, D18Mit68, D18Mit14, D18Mit35, D18Mit123, D18Mit2, D18Mit186, D19Mit109, D19Mit13, D19Mit1, D19Mit71, DXMit55, DXMit166, DXMit114, DXMit172, DXMit153, DXMit186*, Genomic DNA prepared from the tail of B6xKJR F_2 _mice was amplified by polymerase chain reaction(PCR) with primer sets for microsatellite markers and analyzed by agarose gel electrophoresis with 4% agarose (3:1 Nusieve:Seakem agarose, FMC Bioproducts, Rockland, ME, USA) in 1 × TAE buffer. The bands visualized by ethidium staining from B6xKJR F_2 _mice were analyzed for length polymorphisms with reference to the polymorphisms of each marker between B6 and KJR.

### QTL analysis

Phenotypic and genetic data for 274 B6xKJR F_2 _progeny mice were analyzed by interval mapping (IM) and multiple regression analysis with R/qtl and J/qtl [26] to detect loci associated with three components of spontaneous home-cage activity. To explore possible pleiotropic effects on the home-cage activity phenotypes, QTL analysis of each trait was also conducted with a correlated phenotype as a covariate. For each analysis, genome-wide thresholds for suggestive (*P *< 0.63), significant (*P *< 0.05) and highly significant (*P *< 0.001) QTL were determined by 1000 permutations.

### Composite interval mapping performed with QTL Cartographer

Composite interval mapping (CIM, [27,28]) was performed with QTL Cartographer ver. 2.0 [29] to study whether multiple QTL exist on the same chromosome where significant QTL were found in the interval mapping analysis. Five markers determined by a forward regression model were used as a background control in a CIM standard model. The window size was 10 cM. To obtain a more reliable threshold value, and to confirm the significance of detected QTLs, one thousand permutation tests (*P *< 0.05) were performed with QTL Cartographer for each trait. Furthermore, the additive effect (E_A_) and dominance effect (E_D_) at the peak of each significant QTL were also calculated by QTL Cartographer to study the effective size of each detected QTLs. Phenotypic variance (V_P_) explained by each QTL was calculated using these values as follows:



Then, the percent contribution of each locus was calculated from the phenotypic variance at the peak of each *Hylaq *locus (V_P_) divided by total phenotypic variance in B6xKJR F_2 _(V_T_).

### Structural equation model (SEM)

In order to characterize the structure of genetic system on spontaneous activity, we applied SEM analysis [30,31] to our data. SEM is a statistical method for testing an assumed causal relationship among variables, and usually represents the relationship as a direct path graph. Thus, we can easily test a model of the influence of the detected QTLs to the phenotype through this analysis. SEM can graphically illustrate the network among multiple genetic and phenotypic factors for spontaneous activity. We conducted SEM analysis by a method reported previously [32] using AMOS 7.0 [33]. In order to conduct SEM analysis, we used QTLs identified by IM for AT and AA, and the identified QTLs by IM with covariates for the phenotypes. Then the path model was tested for the genetic model of QTLs for two quantitative traits, AT and AA.

An initial path model was defined based on an instruction by Li et al. [32] and the model was refined by following two steps.

First step: Path was deleted when the t-test value was not significantly different from zero or path coefficient with absolute value was lower than 0.05. In this case, change of the chi-square goodness of fit statistic should be less than 3.84 to indicate insignificance in the likelihood ratio test. If the obtained model meets following six standards [32], then the model was considered as a final path model. (1) it should be identified or overidentified with at least 1 residual df; (2) the goodness-fit test should be *P *> 0.05; (3) the largest standardized residual should not exceed 2.0 in absolute value; (4) individual path coefficients should be significantly different from zero based on the t-test; (5) standardized path coefficients should not be trivial (absolute values exceed 0.05); (6) a substantial proportion of the phenotypic variance of the endogenous variables should be explained by the model. If any of these standards were not fit to the model, we further modified as described in the Second step.

Second step: Add a path which causes a biggest change in p-value for the chi-square goodness of fit test and a significant change in the likelihood ratio test. Then, the modified model was tested as described in the First step.

### Statistical analyses

Data analysis was performed with the StatView software package (SAS Institute Inc., Cary, NC, USA) and SPSS software package (SPSS Inc., Chicago, USA). Analysis of variance (ANOVA) was applied to determine the effect of strain or genotype. Post hoc analysis utilized the Fisher's Protected Least Significant difference (PLSD) test. The Kolmogorov-Smirnov (KS) test [34] was used to test the normality of the distribution on the measurements. In the KS test, when KS value exceeded 0.05, normal distribution model will not be denied.

### Data submission

Mapping information of identified QTLs, *Hylaq1*, *Hylaq2 *and *Hylaq3 *have been deposited in the Mouse Genome Database [35].

## Results

### Spontaneous home-cage activity

**Figure 1:**
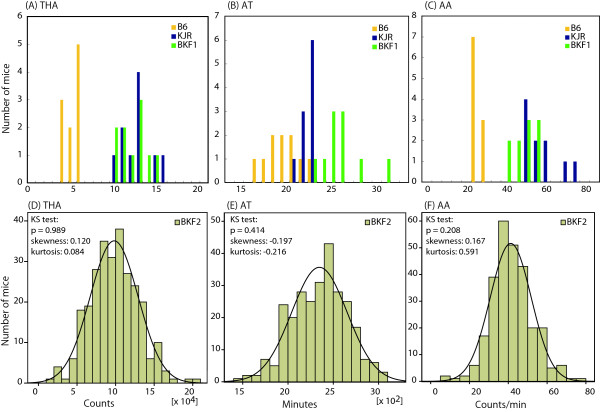
**Histogram and approximated curve to normal distribution of THA, AT and AA, B6xKJR F_1 _and B6xKJR F_2 _progeny**. (A), (B), (C): Distribution of scores in B6, KJR, and B6xKJR F_1_. (D), (E), (F): Distribution of scores in B6xKJR F_2 _population. Normality was assessed by KS test.

Distributions in the parental strains and B6xKJR F_1 _for THA are shown in Figure 1(A). The groups were too small to conduct a meaningful assessment of the distribution but on the basis of the distributions of the F_2 _data (see below) we used parametric statistics for these analyses. One-way ANOVA indicated a significant difference among mouse groups in total home-cage activity (THA), F (2,28) = 80.3, *P *< 0.0001 (Table 1). KJR and B6xKJR F_1 _groups had significantly greater THA than B6 (post hoc analysis, *P *< 0.0001 in both cases). There was no significant difference between KJR and B6xKJR F_1 _groups.

**Table 1:** Comparison of the spontaneous home-cage activity in the parental strains, B6xKJR F_1 _and B6xKJR F_2_

	Average value				
		
	C57BL/6J	KJR	B6xKJR F_1_	B6xKJR F_2_	H^2^
THA	46.5 (8.9)	122.3 (15.9)*	120.5 (19.1)*	98.0 (31.0)	0.62
AT	19.6 (1.8)	22.1 (0.9)*	25.7 (2.1)**	23.5 (3.1)	0.53
AA	23.6 (3.2)	55.8 (6.2)*	46.9 (5.6)*	41.2 (10.6)	0.72

Number of female mice in the home-cage activity study: B6, N = 10; KJR, N = 10; B6xKJR F_1_, N = 11; B6xKJR F_2_, N = 274. THA, total home-cage activity (× 10^3^); AT, active time (× 10^2^); AA; parentheses indicate standard deviation; broad-sense heritability H^2 ^is calculated as follows; H^2 ^= (Variance of B6xKJR F_2 _- Variance of B6xKJR F_1_)/Variance of B6xKJR F_2_. Asterisks indicate significant difference between B6 and KJR or B6 and B6xKJR F_1_. Double asterisk indicates significant difference between KJR and B6xKJR F_1_.

THA was divided into two components, active time (AT) and average activity (AA). AT, an index of 'temporal component' was calculated as total minutes that exhibited more than one count within a one-minute interval. Hence, AT was estimated as the approximate duration of movement. Average activity (AA), an index of 'intensity' was calculated as follows:



The AA reflected average amount of locomotion over one minute of active time.

Distributions of the parental strains and B6xKJR F_1 _for AT and AA are shown in Figure 1B and 1C. One-way ANOVA found a significant difference among different groups of mice in AT, F (2, 28) = 34.3, *P *< 0.0001 (Table 1). KJR showed 13% higher AT value than B6 (post hoc analysis, *P *< 0.01). The AT of the B6xKJR F_1 _population was significantly greater than that of B6 and KJR (post hoc analysis, *P *< 0.0001).

In AA, one-way ANOVA indicated significant mouse group difference, F (2, 28) = 76.2, *P *< 0.0001 (Table 1). KJR exhibited more than 2-fold greater AA than B6 (post hoc analysis, *P *< 0.0001). The AA of B6xKJR F_1 _was intermediate between KJR and B6: AA in B6xKJR F_1 _was significantly higher than that of B6 (*P *< 0.0001) and lower than that of KJR (*P *< 0.01).

### The relationship among three traits associated with home-cage activity

The Kolmogorov-Smirnov (KS) values (Figure 1D, E, F) were consistent with normality for all three indices (THA, AT and AA) and hence we used parametric statistics. In order to test the relationship between the three variables in the B6xKJR F_2 _we conducted Pearson's correlation test (Additional file 1). THA and AT were moderately associated (r = 0.686, *P *< 0.0001), whereas THA and AA showed a high correlation (r = 0.935, *P *< 0.0001). And AA and AT were modestly correlated (r = 0.401, *P *< 0.0001). Thus the outcome of analysis suggested that mechanisms underlying variation of the AA and THA variables have a greater degree of commonality than those underlying variation between the AT and THA.

### QTL analyses

**Figure 2:**
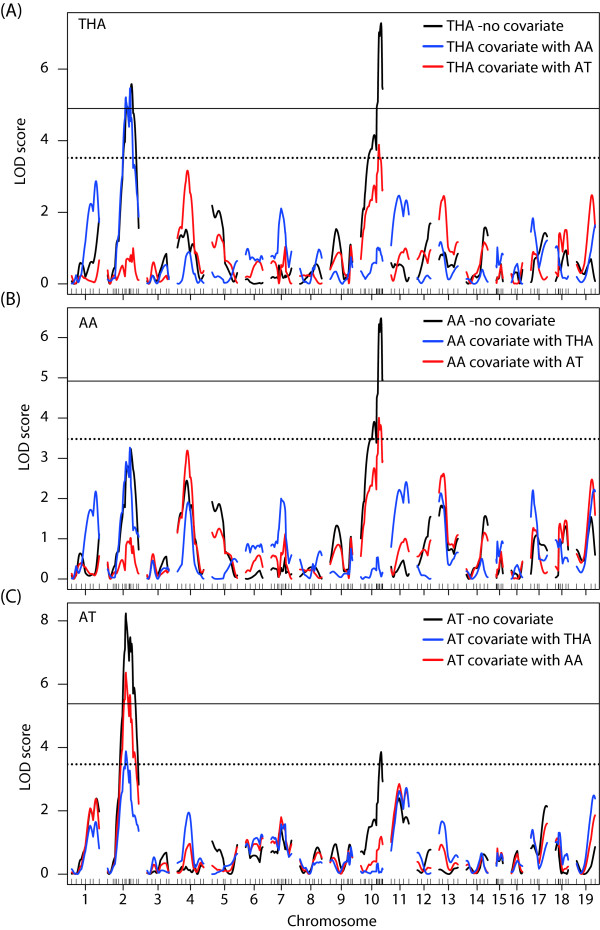
**Results of Interval mapping (IM)**. IM on (A) THA with no covariate (black line), and THA with AT (dashed line) or AA (thin line) as a covariate, (B) AA with no covariate (black line), and AA with AT (dashed line) or THA (thin line) as a covariate, and (C) AT with no covariate (black line), and AT with AA (dashed line) or THA (thin line) as a covariate. Upper and lower horizontal lines in (A), (B) and (C) indicate levels of highly significant, and significant LOD scores, respectively.

Broad-sense heritability values (H^2^) in THA, AT, and AA were 0.62, 0.53 and 0.72, respectively (Table 1). In order to identify the genetic factors related to these traits, we conducted QTL analyses. Interval mapping (IM) analyses detected two highly significant QTLs (*P *< 0.001) on Chr 2 and 10 for THA (Figure 2A, black lines), one highly significant QTL on Chr 10 for AA (Figure 2B, black lines), and one highly significant QTLs on Chr 2 and a significant QTL on Chr 10 for AT (Figure 2C, black line). No significant epistatic interaction between markers was detected in an analysis using R/qtl (data not shown).

Since correlation analyses indicated that three traits of spontaneous activity were interrelated with each other, to assess the nature of their relationship we conducted QTL analyses on each trait of spontaneous activity with two other traits as covariates (Figure 2A–C). The results suggested pleiotropic effects of the QTL on Chr 2 (on THA and AT) and on Chr 10 (on THA and AA).

Mice exhibited a remarkable difference of activity between the light and dark phases (Additional file 2). We divided home-cage activity data into two periods, light phase activity and dark phase activity, and conducted QTL analyses to search for loci related to home-cage activities in these two phases. No significant loci were identified in the light phase (Additional file 3), whereas, in the dark phase, we obtained similar results of QTL as that of whole periods (Additional file 4). In order to investigate effect of day of the experiment, we also conducted IM on each of three days activity data. However, we did not observe a clear difference in the genetic architecture on any day (Additional file 5).

**Figure 3:**
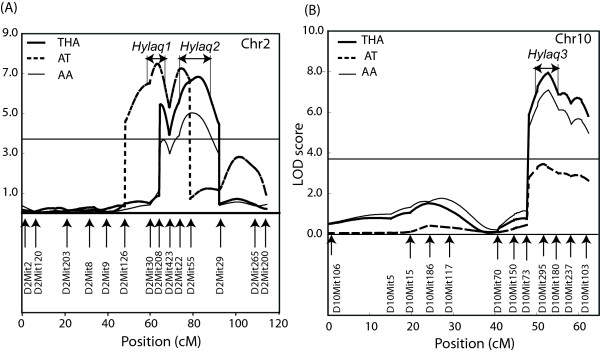
**Results of composite interval mapping (CIM)**. Detection of QTLs for the spontaneous home-cage activity on Chr2 (A) and on Chr10 (B). The genetic markers used for the typing are illustrated in the Figure. The horizontal line indicates significant threshold of LOD (3.7) calculated by permutation test (*P *< 0.05). Two-headed arrows indicate one-LOD drop off confidence intervals.

In order to investigate QTLs on Chr 2 and 10 in more detail, we applied Composite interval mapping (CIM) to these chromosomes. Permutation tests (*P *< 0.05) showed that the threshold values of significant LOD scores were 3.6, 3.7 and 3.6, for THA, AT and AA, respectively. The CIM detected two significant LOD peaks associated with THA, AT, and AA on Chr 2 (Figure 3A, Table 2). Two linked QTL were also implied by a multi-trait analysis on the correlated AT and AA phenotypes (Additional file 6). In the middle region on Chr 2, significant peaks involved in THA, AT and AA overlapped each other and were located between *D2Mit126 *and *D2Mit423*. We designated this locus hyperlocomotor activity related QTL (*Hylaq1*). In the distal side of the *Hylaq1 *on Chr 2, significant peaks associated with THA, AT and AA were located between *D2Mit22 *and *D2Mit29*. This locus was designated *Hylaq2*. In the telomeric region on Chr 10, a broad locus with several peaks associated with THA and AA was detected between *D10Mit73 *and *D10Mit180*. This locus was designated *Hylaq3*, mainly associated with THA and AA (Figure 3B, Table 2). Scores of effect size showed a relatively high contribution to the phenotypic variance (Table 2): *Hylaq1 *and *Hylaq2 *accounted for 11.9 and 15.2%, respectively, on AT.

**Table 2:** QTL for spontaneous home-cage activity

QTL	Chr.	Markers (position (cM))		Trait	LOD	E_A_	E_D_	Contribution (%)
							
		Proximal	Distal					
*Hylaq1*	2	*D2Mit126 *(48.5)	*D2Mit423 *(69.1)	THA	5.4*	8.6 × 10^3^	5.3 × 10^3^	4.5
				AT	7.5*	1.5 × 10^3^	0	11.9
				AA	3.7*	1.2	2.9	2.5
*Hylaq2*	2	*D2Mit22 *(73.4)	*D2Mit29 *(92.3)	THA	6.8*	12.4 × 10^3^	3.3 × 10^3^	8.9
				AT	7.2*	1.7 × 10^3^	-0.2 × 10^3^	15.2
				AA	5.0*	2.2	2.4	3.6
*Hylaq3*	10	*D10Mit73 *(48.6)	*D10Mit180 *(56.2)	THA	7.9*	13.5 × 10^3^	0.4 × 10^3^	9.5
				AT	3.4	1.1 × 10^3^	0	6.4
				AA	7.1*	4.0	1.0	7.3

QTLs for spontaneous home-cage activity are shown. Markers indicate flanking microsatellite markers located at just outside of one LOD support interval of the peaks for AT or THA. Position indicates genetic distances from centromere to marker in a scale of cM. The marker positions were obtained from the genetic distance calculated by the recombination frequency of B6xKJR F_2_. The peak LOD score at *Hylaq1 *and *Hylaq2 *were calculated by QTL Cartographer. Asterisks indicate significant QTLs which were above scores obtained by permutation tests (THA: 3.6, AT: 3.7 AA: 3.6). E_A _and E_D _indicate the additive effect and dominance effect, respectively, that were calculated by QTL Cartographer. Contribution were calculated by phenotypic variance at the peak of each QTL (*V*_*P *_= *E*_*A*_^2^/2 + *E*_*D*_^2^/4) divided by total phenotypic variance (V_T_) in B6xKJR F_2_.

**Figure 4:**
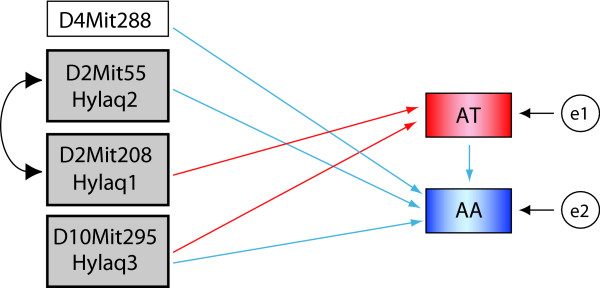
**Final path illustrations obtained by structural equation model analysis (SEM)**. SEM analysis of two components, AA and AT, of spontaneous home-cage activity. e1 and e2 indicate the unobserved residual error. *D4Mit288 *is a locus which showed no significant LOD acore in the IM analysis. A curved arrow indicate genetic linkage between two loci.

In order to explore the relationship among the QTL affecting different activity components, AT and AA, and also the relationship between the two components themselves we conducted SEM analysis based on the results of IM with covariates (Figure 4). The results (for details see Methods and Additional file 7) indicated that both *Hylaq1 *and *Hylaq3 *influence AT and both *Hylaq2 *and *Hylaq3 *associate with AA, furthermore, it showed that variation in AT was causative to that in AA.

## Discussion

In this study, we investigated the difference in spontaneous locomotor activity in terms of total home-cage activity, by partitioning it into temporal and velocity elements of the behavior, between the KJR and B6 strains. Pearson correlation analysis between AT and AA showed a low score (r = 0.400), indicating that these two measurements reflect largely distinct biological processes, AT is temporal (how much time from the total period of 72 hours animals were active) whereas AA is the average amount of activity within a 1 minute period. A high correlation between AA and a collective total home cage activity, THA, (0.935) indicated that the intensity of activity within 1 min time period predicted the total activity across 3 days. Partitioning of THA into AA and AT provided information about the effects of the genetic variation on the processes contributing to the overall spontaneous activity. This is consistent with a previous study which also demonstrated that a 'temporal element' and 'velocity' in a home-cage are controlled by different genetic bases [19].

QTL analysis identified three loci, *Hylaq1*, *Hylaq2 *and *Hylaq3*, associated with spontaneous home-cage activity. These QTLs, especially, *Hylaq1 *and *Hylaq3 *have a relatively large effective size (11.9 and 15.2% of phenotypic variance, respectively; Table 2), which exceed generally found effects in behavioral traits [36], and will facilitate fine mapping of these loci. By searching the MGI database [35], a number of locomotor activity-related QTLs were found in the region between *D2Mit126 *and *D2Mit29 *(Table 3, [6,7,17,37-41]), these markers are located at just outside of two LOD support interval (90% confidence interval) below the peak of *Hylaq1 *and *Hylaq2*. Because the genetic positions were not precisely comparable among different experiments, we only list the following QTLs as possibly having genetic communality with *Hylaq1*, *Hylaq2 *and *Hylaq3*. Five QTLs, *Actre2, Etohr, Cplaq7, Actre3 *and *Actre4*, are located around the region of *Hylaq1 *and four QTLs, *Rrodp2, Slms2, Nilac3 *and *Dloc1 *which are in close proximity to the *Hylaq2 *loci on Chr 2. Concerning *Hylaq3*, there are no QTL directly associated with spontaneous home-cage activity at the same locus on Chr 10 in the database. However, one QTL, *Exq1*, associated with open-field ambulation was reported in the region just distal side of the two LOD support interval markers, *D10Mit73 *and *D10Mit103*, on Chr 10 [3,42].

**Table 3:** QTLs related to locomotor activity overlapping to *Hylaq1 *and *Hylaq2 *region on Chr 2.

QTL	Position (cM)	Phenotype	Reference
*Actre2*	40–60	locomotor activity in response to alcohol challenge compared	[17,27]
*Etohr*	48	locomotor activity in response to alcohol challenge compared	[28]
*Cplaq7*	53	circadian period of locomotor activity	[29,30]
*Actre3*	62–64	locomotor activity at later time intervals following ethanol challenge (10–15 min)	[17]
*Actre4*	64–66	locomotor activity at later time intervals following ethanol challenge (15–20 min)	[17]
*Rrodp2*	74	rotarod performance at day 2	[6]
*Slms2*	80	pleiotropic effects on sensitivity to locomotor stimulants, ethanol and allopregnanolone	[31]
*Nilac3*	83.1	locomotor activity after nicotine administration	[7]
*Dloc1*	84.2	duration of locomotor activity	[6]

In this study, we found that *Hylaq3 *influences both the temporal and intensity elements of spontaneous activity (AT and AA) in a pleiotropic manner. In the context of the phenotype-specific effects of the *Hylaq1 *and *Hylaq2*, this observation suggests an overlap of a certain degree between the mechanisms underlying temporal- and intensity-related phenotypes of spontaneous home cage activity. However, further research is required to discriminate between the scenarios of the pleiotropic effect of the same gene versus the influence of closely linked genes on AT and AA.

In the present study the *Hylaq1, Hylaq2 *and *Hylaq3 *loci were identified in the analysis of the composite THA phenotype, thus partitioning it into the AT and AA components did not identify additional loci. However, this approach provided auxiliary information about the nature of variation in THA, e.g., longer cumulative period of activity *vs *greater intensity, which might be important for nomination of the candidate genes in the fine mapping stage the effort.

In the present study, we used only females to examine home-cage activity, because the previous study showed clear difference of activity of females in the parental strains. However, it is possible that we could find different QTL in males. This attempt to find sex specific QTL on behaviour will be of considerable value.

## Conclusion

The present study clarified the genetic basis for different components of spontaneous activity. The identified QTLs had a relatively large effect size and are amenable for subsequent fine mapping studies. This result support the potential of wild derived strains, such as KJR, in understanding the mechanism of genetic regulation of behavioural phenotypes.

## Authors' contributions

JU collected data for behaviors and genotypes, analyzed the data, and made a draft of the manuscript. AN participated in the behavioral analyses. AL and DAB conducted QTL analysis with covariates. TS and SK conducted SEM analysis. TK supervised the project. All authors read and approved the final manuscript.

## Supplementary Material

Additional file 1 **Correlations between the two measured traits**. Correlations between AT and AA in B6xKJR F_2 _progeny. Click here for file

Additional file 2 **Distribution of activity scores in B6xKJR F2**. Distribution of THA, AT and AA in whole period (white bars) and in dark period (black bars) in B6xKJR F_2_. Click here for file

Additional file 3 **Interval mapping on activity data of the light phase**. No significant QTL was found on activity data of the light phase. Click here for file

Additional file 4 **Results of interval mapping on activity data of the dark phase**. QTLs found on activity data in dark phase were similar to that of whole period. Click here for file

Additional file 5 **Results of interval mapping on each day's data**. Results of QTL analysis on each day's activity data showed similar pattern as entire three days period. Click here for file

Additional file 6 **Multiple traits analysis using AT and AA**. Multiple traits analysis also supports that there are at least two loci, *Hylaq1 *and *Hylaq2*, located close to each other on Chr 2. Click here for file

Additional file 7 **Structural Equation Modeling (SEM) analysis**. The path model indicated that *Hylaq1 *influences AT, *Hylaq2*, AA, while *Hylaq3 *is associated with both AA and AT. Click here for file
