# Supplementary material for: QTL analyses of temporal and intensity components of home-cage activity in KJR and C57BL/6J strains
Source: BMC Genet. 2009 Jul 29;10:40. doi: 10.1186/1471-2156-10-40 (PMC2723135; doi:10.1186/1471-2156-10-40)

Additional file 1 – Correlations between the two measured traits involved in the spontaneous home-cage activity in B6xKJR F2 progeny.
(A) Correlation between AT and THA; r= 0.686, *P* < 0.0001. (B) Correlation between AA and THA; r= 0.935, *P* < 0.0001. (C) Correlation between AT and AA; r= 0.401, *P* < 0.0001.

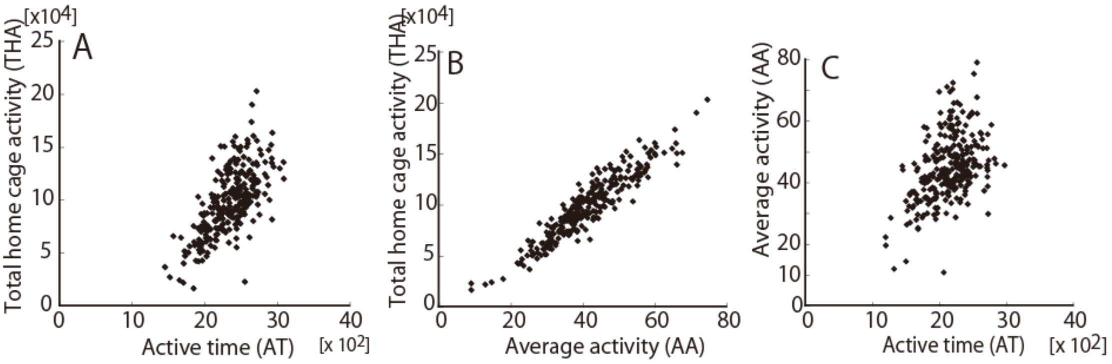

Supplement: Additional file 1 — Correlations between the two measured traits. Correlations between AT and AA in B6xKJR F2 progeny. [file 1471-2156-10-40-S1.pdf]
