# Supplementary material for: QTL analyses of temporal and intensity components of home-cage activity in KJR and C57BL/6J strains
Source: BMC Genet. 2009 Jul 29;10:40. doi: 10.1186/1471-2156-10-40 (PMC2723135; doi:10.1186/1471-2156-10-40)

Additional file 2 – Distribution of activity scores in whole period (white bars) and in dark period (black bars) in B6xKJR F_2. THA, AT and AA in the light phase (9:00-19:00) and the dark phase (21:00-7:00) were calculated separately. We eliminated the data in the periods from 7:00 to 9:00, and from 19:00 to 20:00, because these marginal periods of L/ D phase were influenced by both phases and quite variable among individuals. (A) Distribution of THA scores for total 3 days, dark phase, and light phase. (B) Distribution of AT scores for total 3 days, dark phase, and light phase. (C) Distribution of AA scores for total 3 days, dark phase, and light phase. The AT of whole period indicated normal distribution, while AT in the dark phase apparently lack higher value than 1800 which is maximum minutes (60min x 10h x 3days). This distribution pattern is due to ceiling effect, some progeny of B6xKJR F2 move more than 1800 min throughout the dark period for three days.

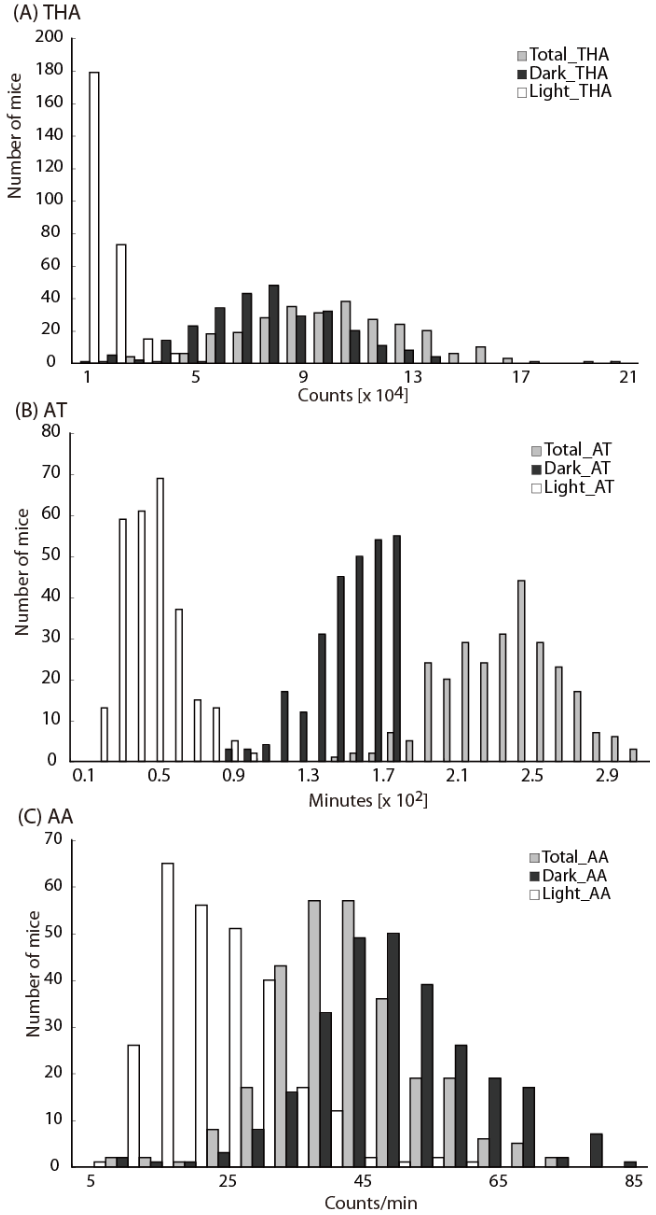

Supplement: Additional file 2 — Distribution of activity scores in B6xKJR F2. Distribution of THA, AT and AA in whole period (white bars) and in dark period (black bars) in B6xKJR F2. [file 1471-2156-10-40-S2.pdf]
