# Supplementary material for: QTL analyses of temporal and intensity components of home-cage activity in KJR and C57BL/6J strains
Source: BMC Genet. 2009 Jul 29;10:40. doi: 10.1186/1471-2156-10-40 (PMC2723135; doi:10.1186/1471-2156-10-40)

Additional file 3 – Results of interval mapping on activity data of the light phase. (A) THA with no covariate (black line), and THA with AT as a covariate (blue line) or AA (red line), (B) AA with no covariate (black line), and AA with AT as a covariate (red line) or THA (blue line), (C) AT with no covariate (black line), and AT with AA as a covariate (red line) or THA (blue line). Significant LOD scores during the light phase were around 3.4 and none of QTL exceeded the significant level.

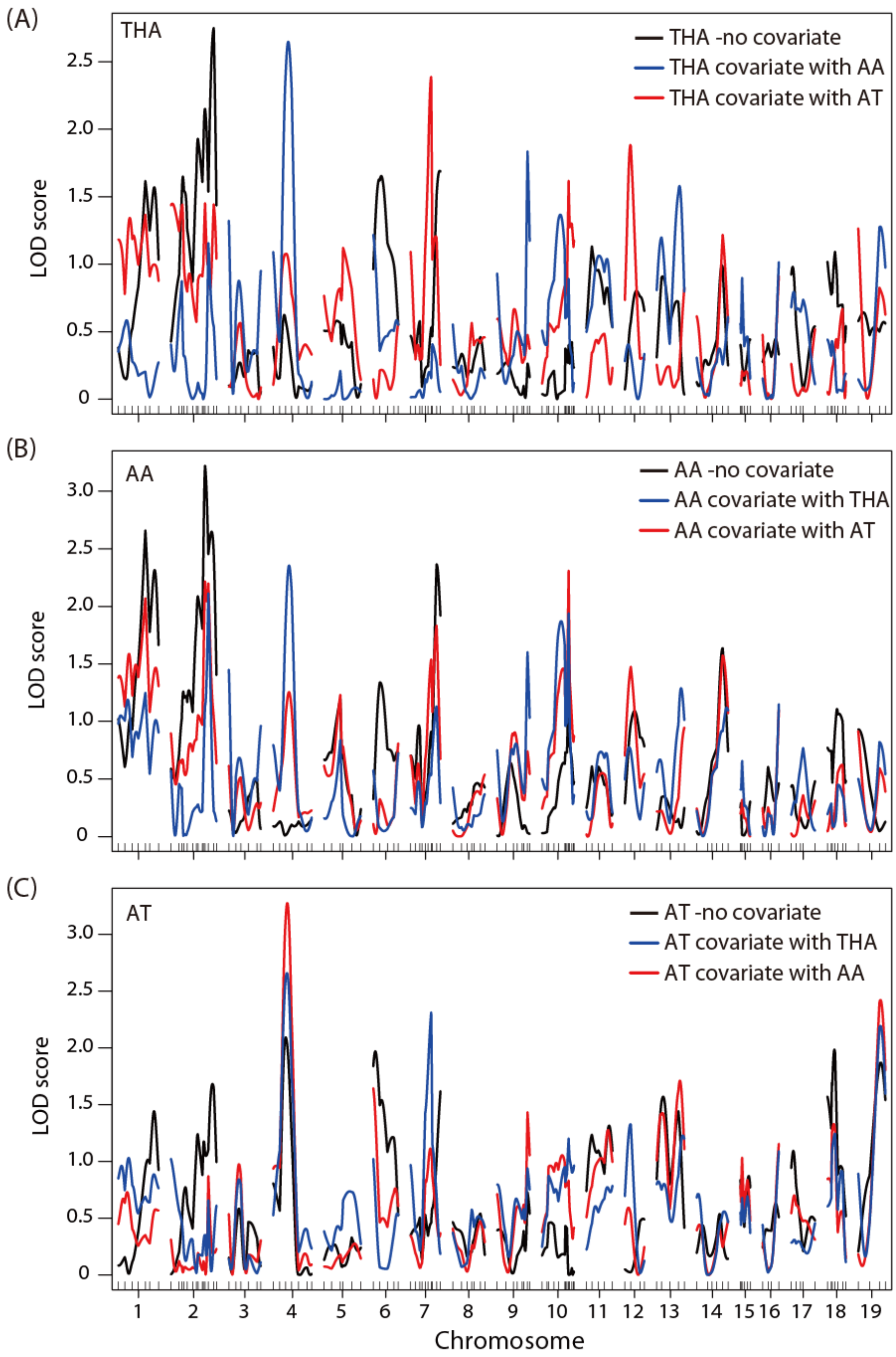

Supplement: Additional file 3 — Interval mapping on activity data of the light phase. No significant QTL was found on activity data of the light phase. [file 1471-2156-10-40-S3.pdf]
