# Supplementary material for: QTL analyses of temporal and intensity components of home-cage activity in KJR and C57BL/6J strains
Source: BMC Genet. 2009 Jul 29;10:40. doi: 10.1186/1471-2156-10-40 (PMC2723135; doi:10.1186/1471-2156-10-40)

Additional file 4 – Results of interval mapping on activity data of the dark phase. (A) THA with no covariate (black line), and THA with AT as a covariate (red line) or AA (blue line), (B) AA with no covariate (black line), and AA with AT (red line) or THA (blue line) as a covariate, (C) AT with no covariate (black line), and AT with AA (red line) or THA (blue line) as a covariate. Upper and lower horizontal lines in (A), (B), and (C) indicate levels of highly significant and significant LOD scores, respectively.

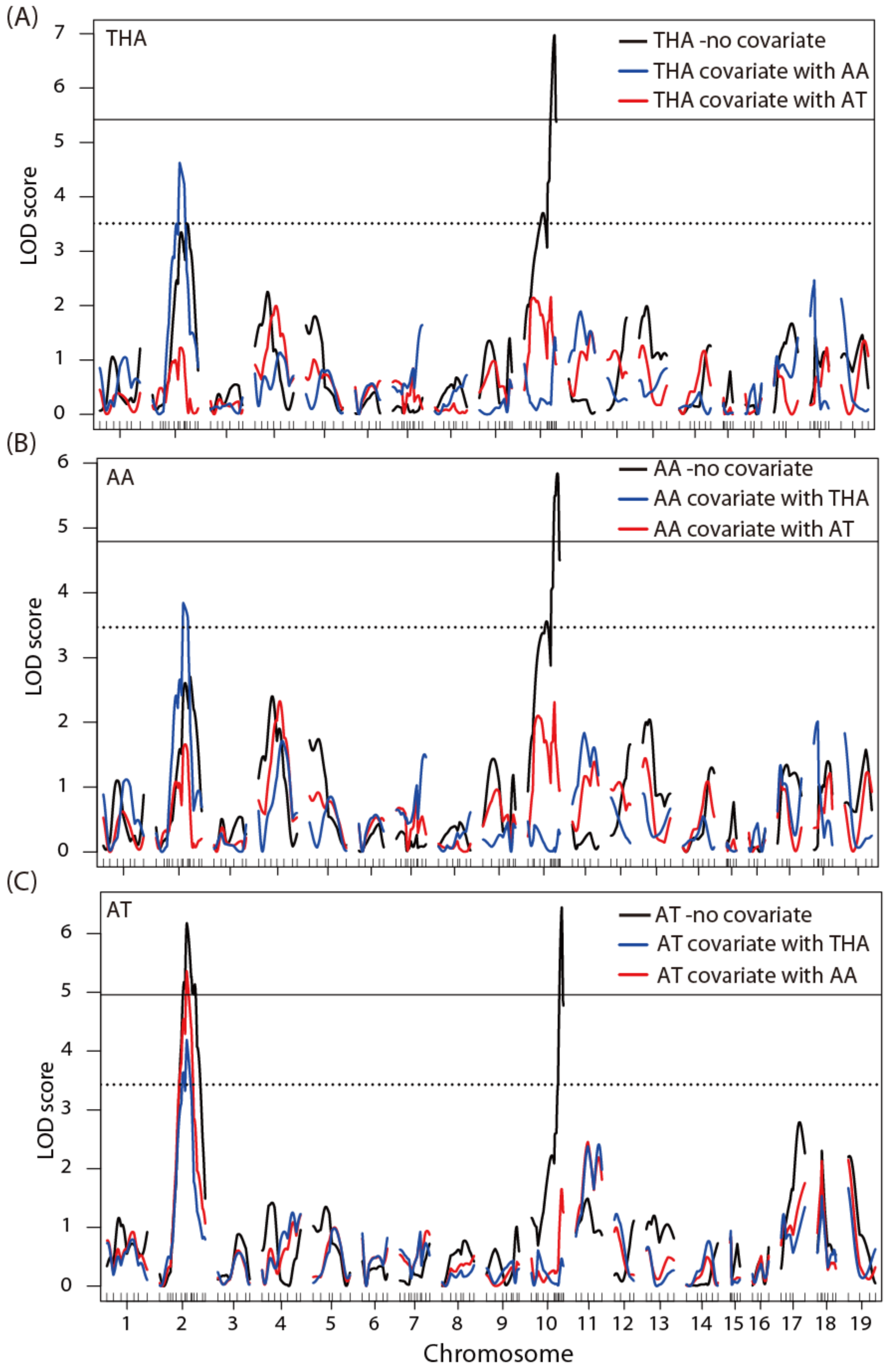

Supplement: Additional file 4 — Results of interval mapping on activity data of the dark phase. QTLs found on activity data in dark phase were similar to that of whole period. [file 1471-2156-10-40-S4.pdf]
