# Supplementary material for: QTL analyses of temporal and intensity components of home-cage activity in KJR and C57BL/6J strains
Source: BMC Genet. 2009 Jul 29;10:40. doi: 10.1186/1471-2156-10-40 (PMC2723135; doi:10.1186/1471-2156-10-40)

Additional file 5 – Results of interval mapping on each day's data.
Results of QTL analysis on each day's activity data showed similar pattern as entire three days period. Genome wide threshold for significant (P<0.05) LOD scores were determined by 1000 permutations for each phenotype.

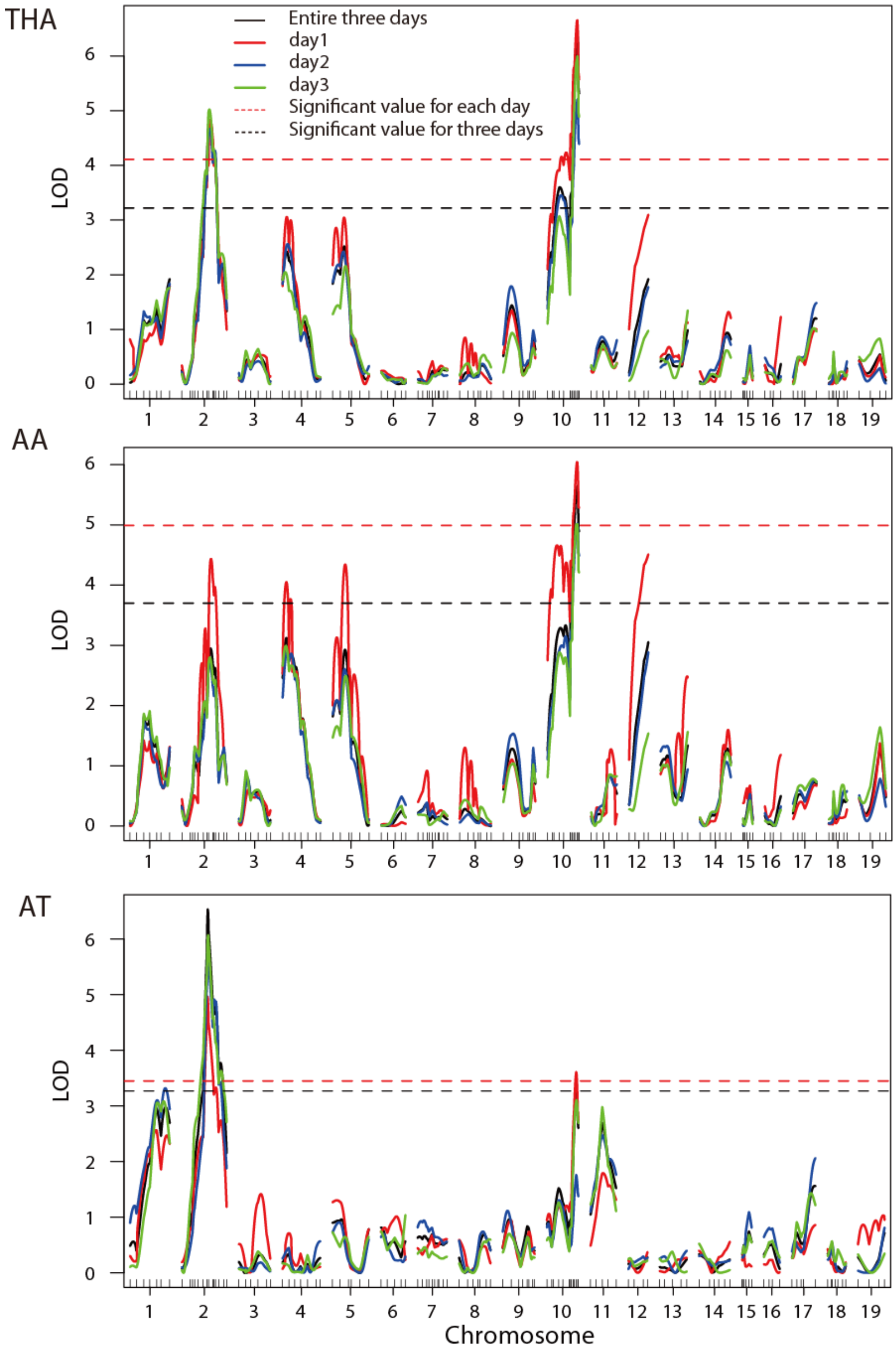

Supplement: Additional file 5 — Results of interval mapping on each day's data. Results of QTL analysis on each day's activity data showed similar pattern as entire three days period. [file 1471-2156-10-40-S5.pdf]
