# Supplementary material for: QTL analyses of temporal and intensity components of home-cage activity in KJR and C57BL/6J strains
Source: BMC Genet. 2009 Jul 29;10:40. doi: 10.1186/1471-2156-10-40 (PMC2723135; doi:10.1186/1471-2156-10-40)
Supplement: Additional file 6 — Multiple traits analysis using AT and AA. Multiple traits analysis also supports that there are at least two loci, Hylaq1 and Hylaq2, located close to each other on Chr 2. [file 1471-2156-10-40-S6.pdf]

Additional file 6 – Multiple traits analysis using AT and AA.
Multiple traits analysis also supports that there are at least two loci, *Hylaq1* and *Hylaq2*, located close to each other on Chr 2. The joint-trait showed two peaks corresponding to the peaks of AT and AA. Another significant peak was appeared at the distal side of these peaks.

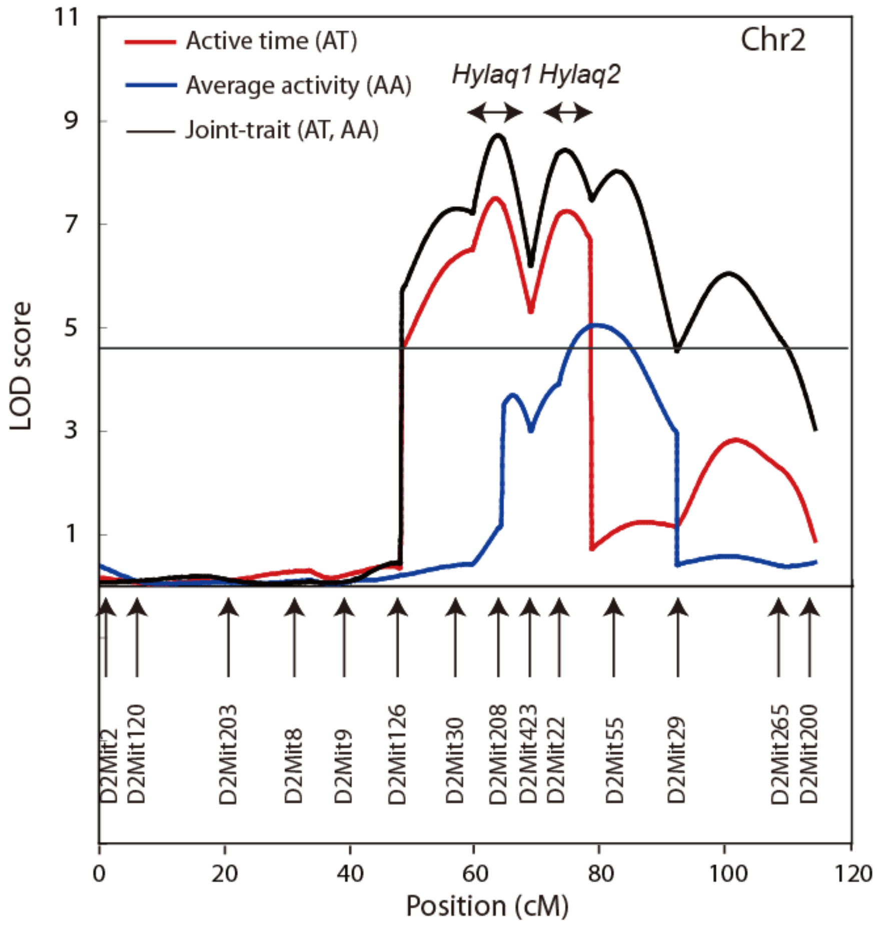

Method: Multiple traits analysis was conducted using QTL Cartograher version 2.5. AT and AA were used as multiple traits. Genome-wide thresholds for significant ($P<0.05$) QTLs were determined based on 1,000 permutations.
