# Supplementary material for: QTL analyses of temporal and intensity components of home-cage activity in KJR and C57BL/6J strains
Source: BMC Genet. 2009 Jul 29;10:40. doi: 10.1186/1471-2156-10-40 (PMC2723135; doi:10.1186/1471-2156-10-40)
Supplement: Additional file 7 — Structural Equation Modeling (SEM) analysis. The path model indicated that Hylaq1 influences AT, Hylaq2, AA, while Hylaq3 is associated with both AA and AT. [file 1471-2156-10-40-S7.pdf]

Additional file 7 – Structural Equation Modeling (SEM) analysis.
In this analysis, THA was not suitable as one of the components because of the confounding effect of these three measures from a statistical point of view as multiplying AT by AA gives THA.

A path model made according to the criteria of a previous report [40] is shown in Figure 4. Five measures of model fit statistics, AIC (Akaike's information criterion), the chi-square goodness of fit statistic (Degree of freedom), CFI (the comparative fit index [43]), RMSEA (the root mean square error of approximation [44]), and the largest standardized residual are shown in below. All values were calculated by AMOS 7.0.

AIC	chi-square(Df)	CFI	RMSEA	standardized residual
45.720	7.720(8)	1.0	0	0.072

It should be noted that CFI > 0.95 or RMSEA < 0.05 are typically considered to indicate a good fit for the model.

Because the first path model satisfied the criteria (Materials and methods), this model was confirmed as a final path model (Fig. 4). The results indicated that *Hylaq1* influences AT, *Hylaq2*, AA, while *Hylaq3* is associated with both AA and AT. This analysis complemented our IM findings by showing that variation in AT was causative to that in AA.

Additional references

43. Bentler PM: **Comparative fit indexes in structural models.** *Psychol Bull* 1990, **107**:238-246

44. Browne MW, Cudeck R: **Alternative ways of assessing model fit.** In: *Testing Structural equation models.* Edited by Bollen KA, Long JS. Sage Publications 1993, 136-162
